# Supplementary material for: The Role of Counterions in the Assembly of Charged Virus-Like Shells
Source: J Phys Chem B. 2025 Sep 12;129(38):9633–42. doi: 10.1021/acs.jpcb.5c03361 (PMC12478857; doi:10.1021/acs.jpcb.5c03361)
Supplement: Supplementary file 1 [file jp5c03361_si_001.pdf]

Supporting Information for

The Role of Counterions in the Assembly of Charged Virus-Like Shells

Ya-Wen Hsiao[1], Magnus Hedström[2], Maxim G Ryadnov[3,4], David J Bray[1], and Jason Crain[5,6]*

[1] The Hartree Centre, STFC Daresbury Laboratory, Warrington, WA4 4AD, UK

[2] Clay Technology, Ideon Science Park, SE-223 70 Lund, Sweden

[3] National Physical Laboratory, Hampton Road, Teddington, TW11 0LW, UK

[4] Department of Physics, King's College London, Strand Lane, London, WC2R 2LS, UK

[5] IBM Research Europe, Hartree Centre, Daresbury, WA4 4AD, UK

[6] Department of Biochemistry, University of Oxford, Oxford OX1 3QU, UK

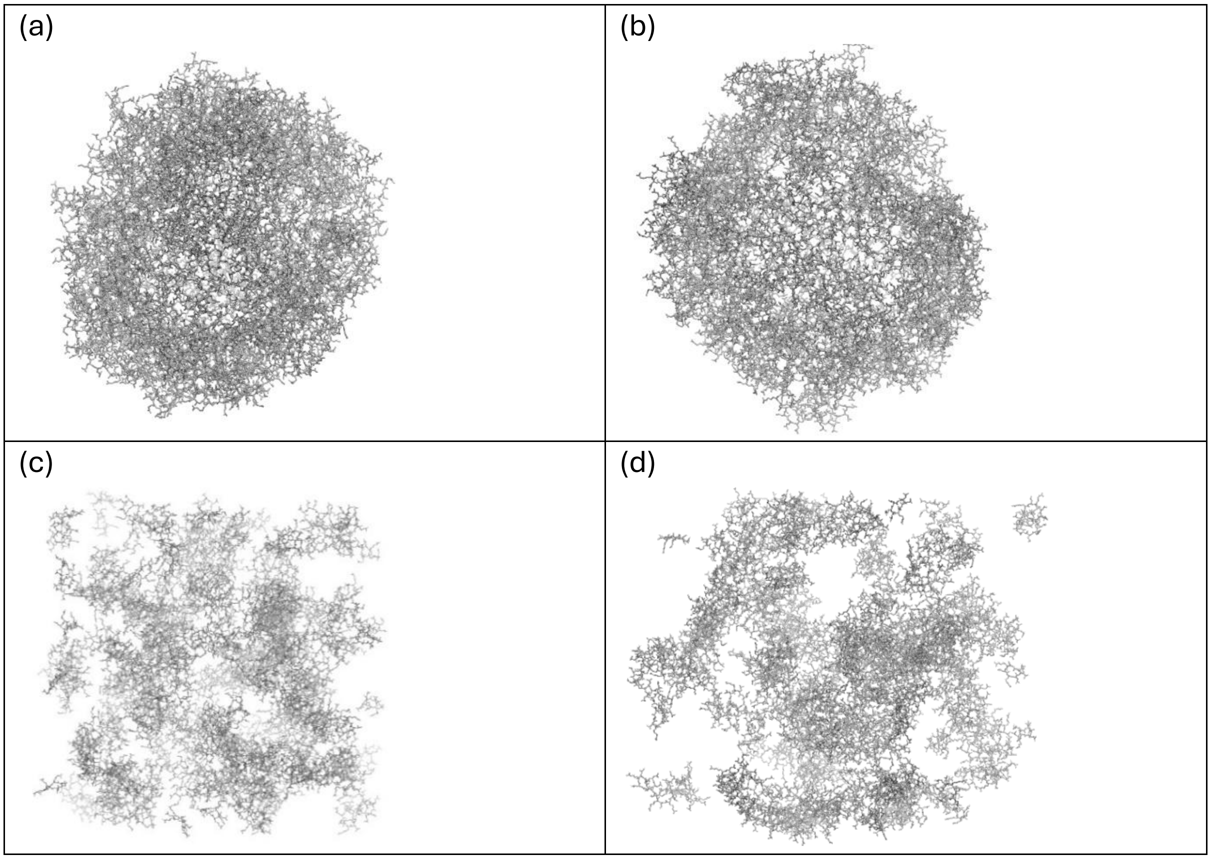

Figure S1. Final configuration of VLP assembly with counterion (a) $H_2PO_4^-$, (b) PO_4^{3-}, (c) Cl^-, (d) F^-

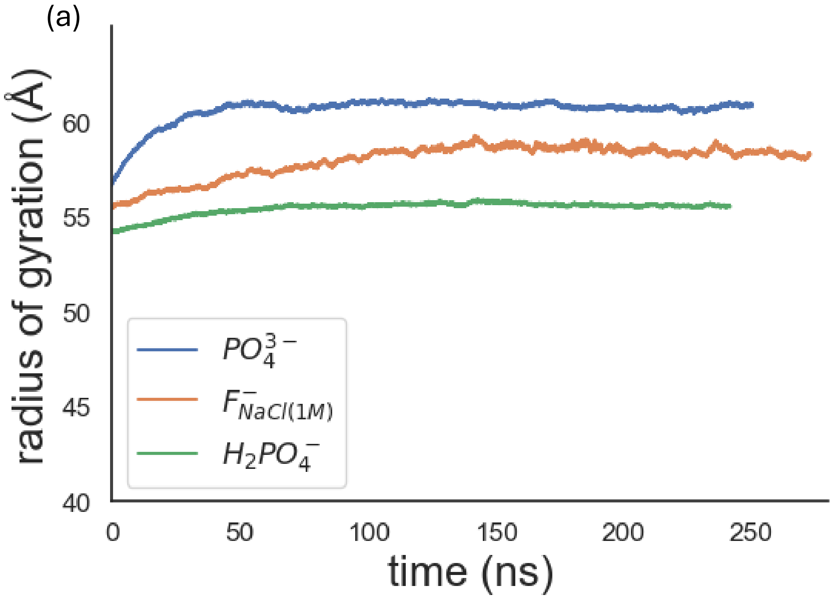

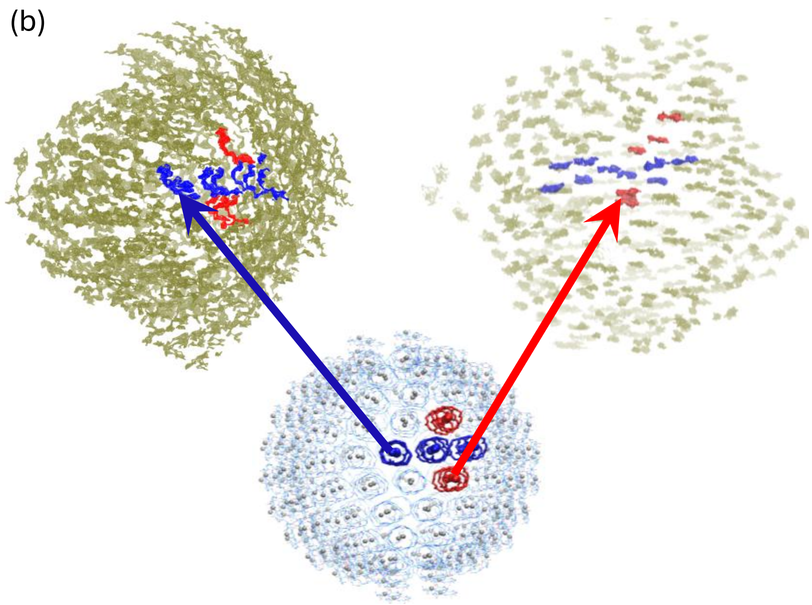

Figure S2. (a) Plateaued radii of gyration indicating convergence; (b) Superposition of the center-of-mass of each peptide, collected every 500 ps over the final 40 ns, in the $H_2PO_4^-$ (upper left) and PO_4^{3-} (upper right) systems, showing that the peptides do not undergo major positional rearrangement but retain the same neighboring relationship as the initial configuration, exemplified by the selected colored stacks compared to the corresponding stacks in the initial packing (lower center). For clarity, only two example arrows are drawn to indicate the positional similarity.

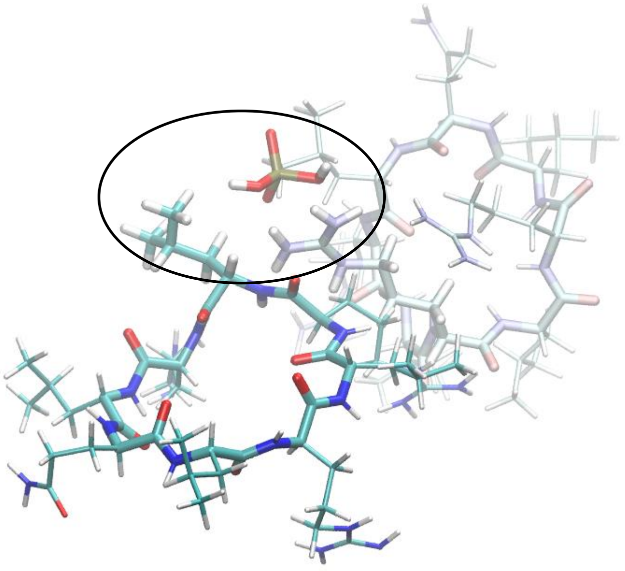

Figure S3. Representative snapshot of $H_2PO_4^-$ in contact with D-Leu through hydrophobic interaction while bridging to an Arg of a neighbor peptide.

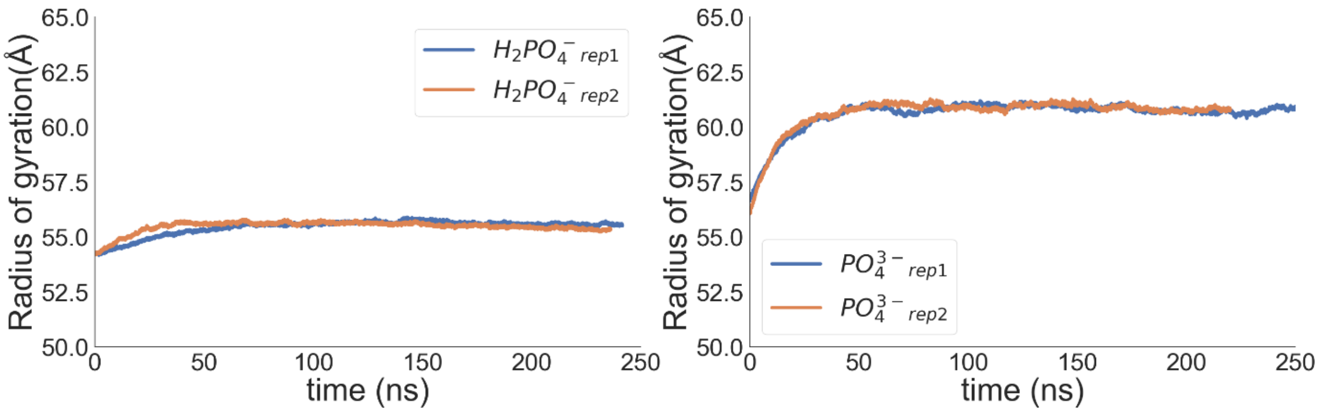

Figure S4. Radius of gyration ($H_2PO_4^-$ and PO_4^{3-})

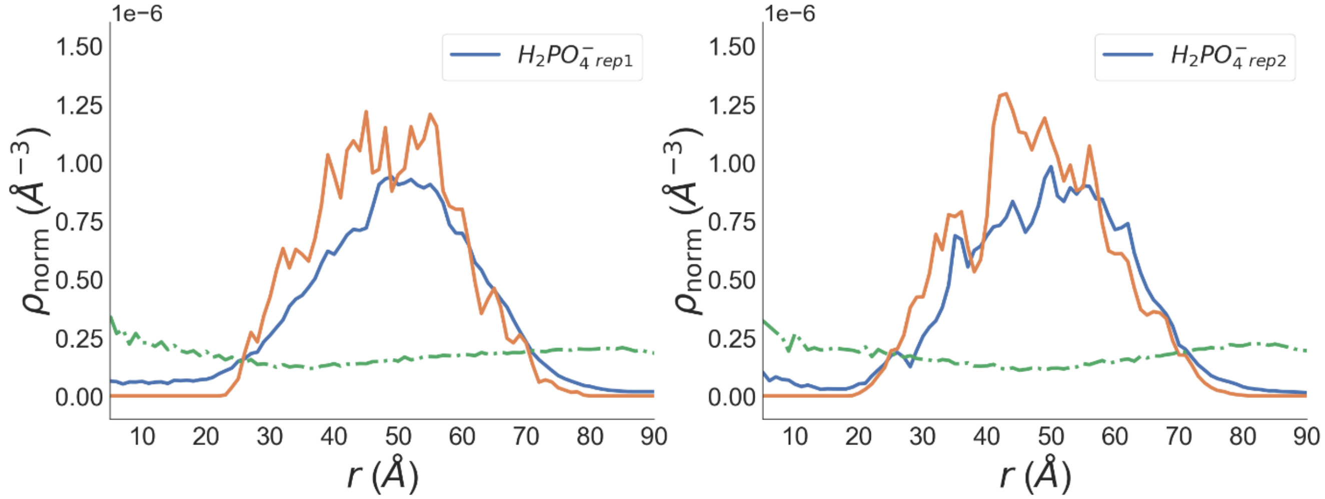

Figure S5. $H_2PO_4^-$: normalized radial number density $\rho_{norm}(r)$ of counterions (blue), peptides (orange), and water (green)

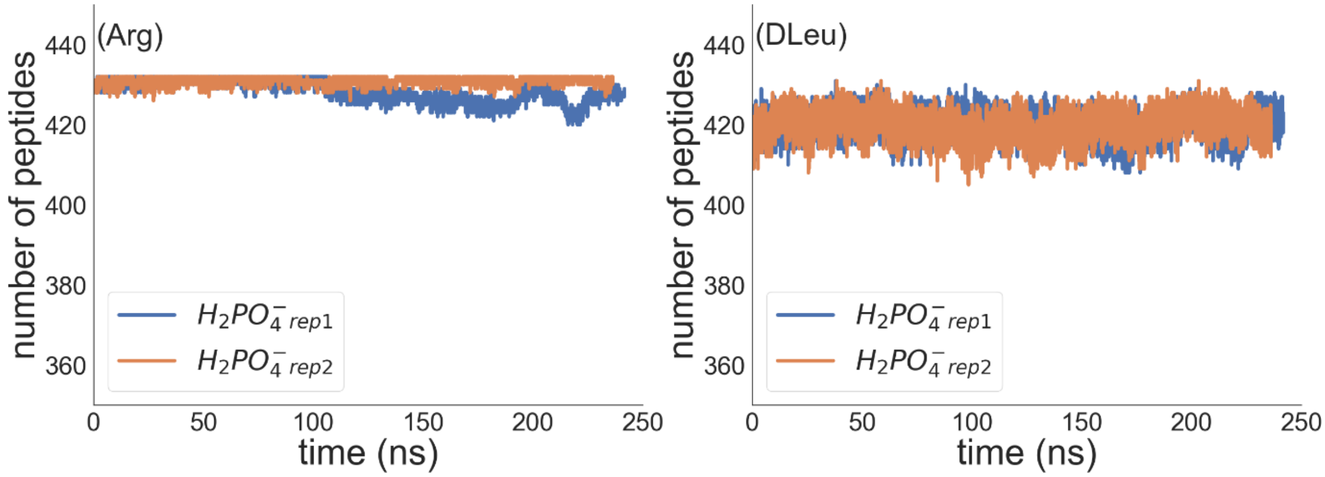

Figure S6. $H_2PO_4^-$: number of peptides whose side chains of Arg (a) D-Leu (b) are in contact with counterions.

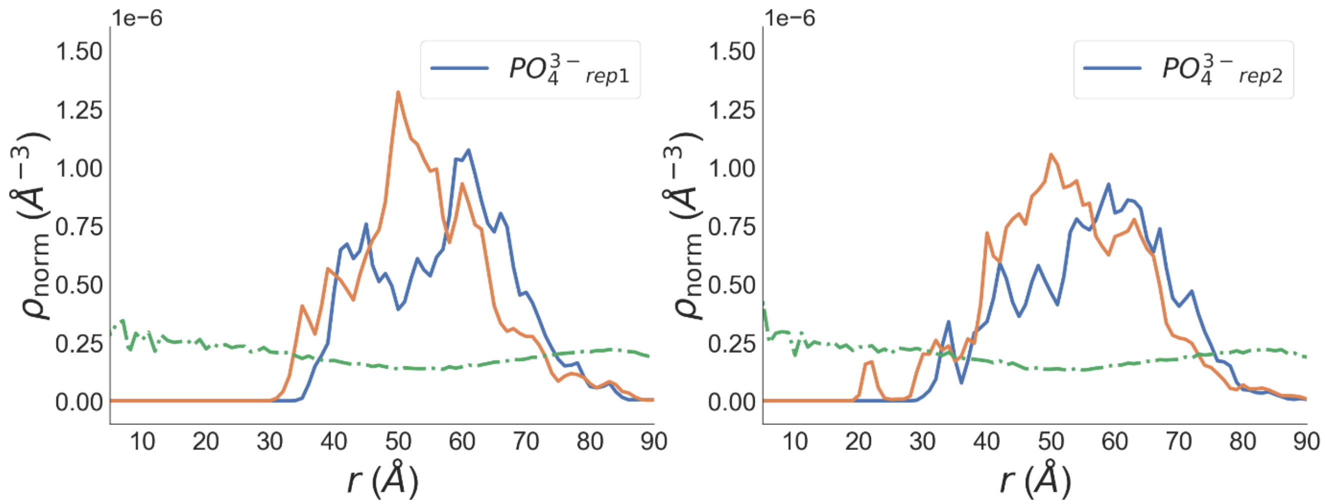

Figure S7. PO_4^{3-}: normalized radial number density $\rho_{\text{norm}}(r)$ of counterions (blue), peptides (orange), and water (green)

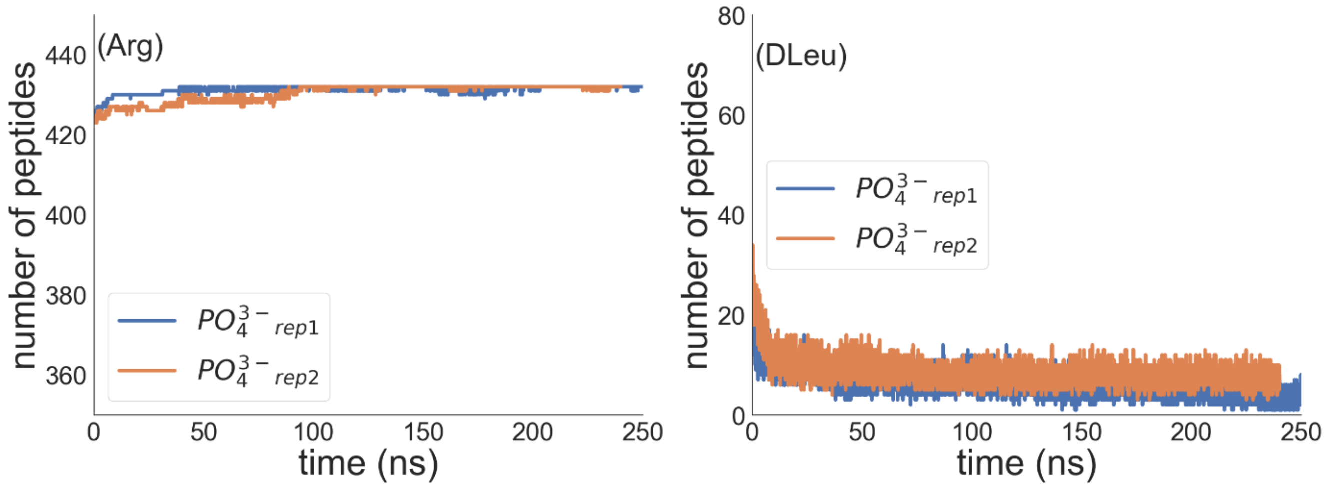

Figure S8. PO_4^{3-}: number of peptides whose side chains of Arg (a) D-Leu (b) are in contact with counterions

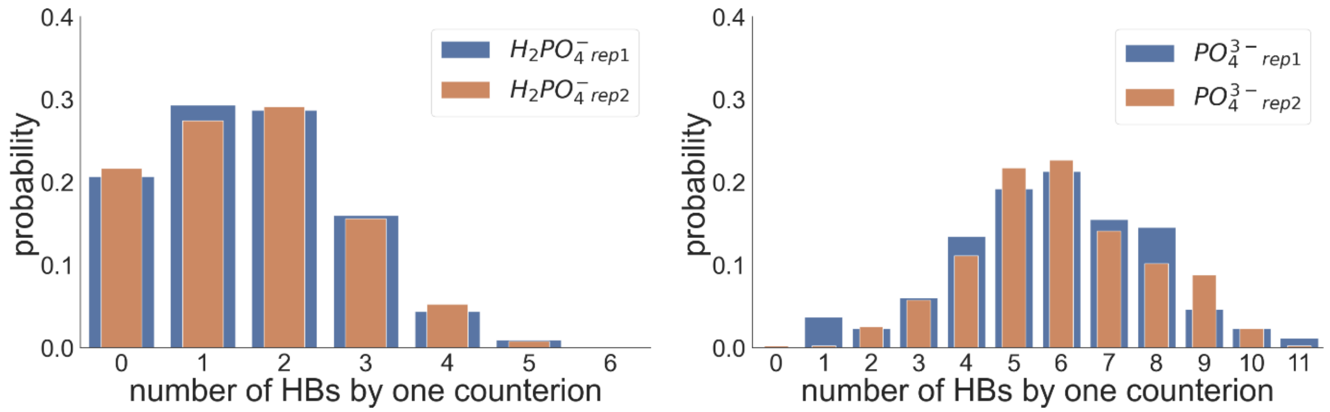

Figure S9. Normalized probabilities (per anion) of the number of hydrogen bonds formed between each counterion and peptides.

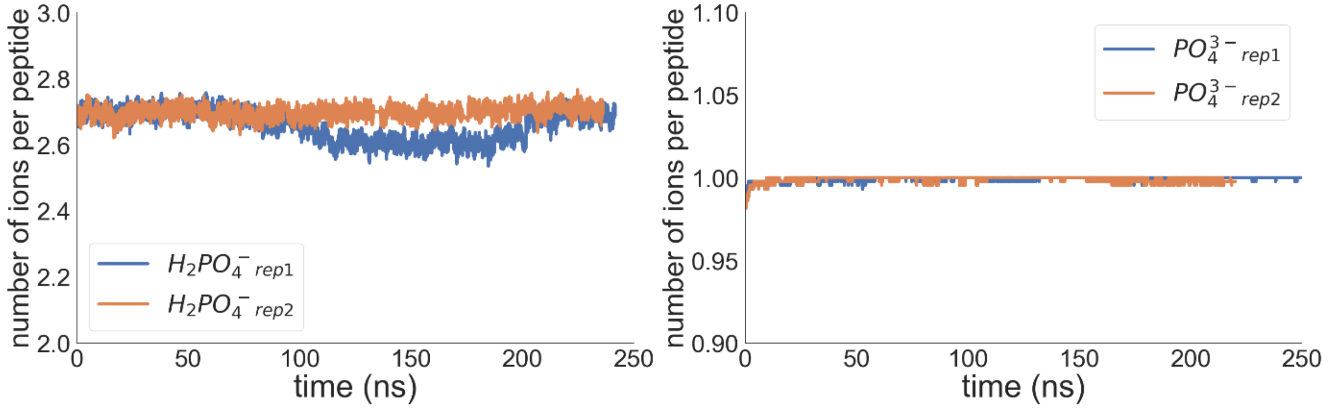

Figure S10. Time evolution of the number of condensed counterions per peptide.

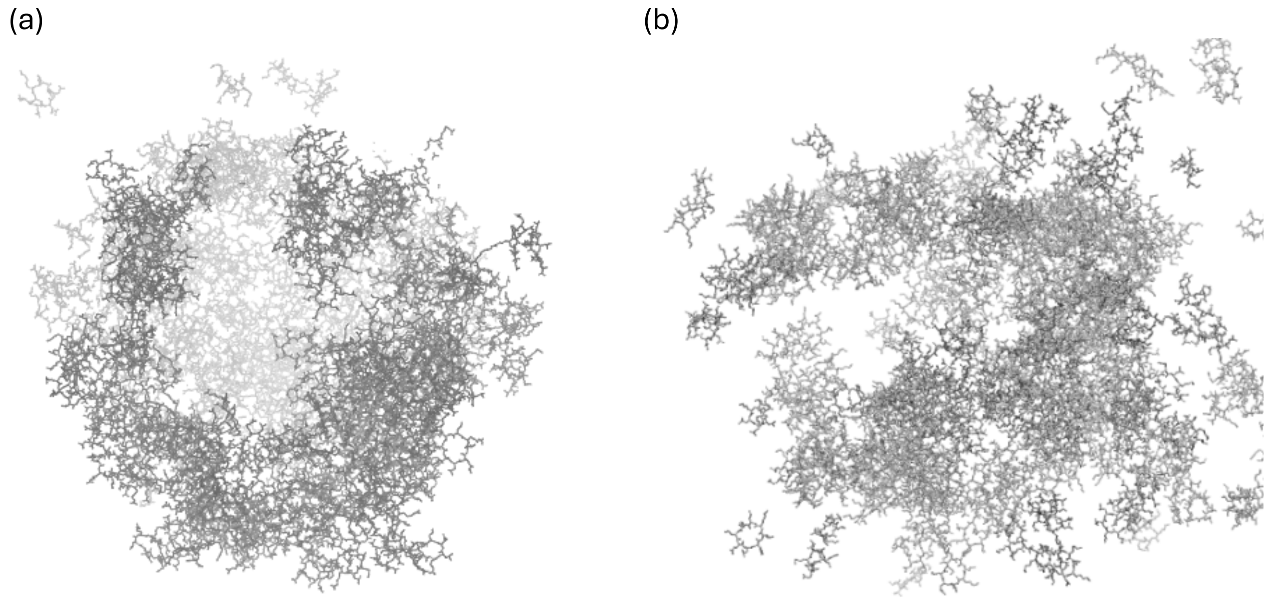

Figure S11. Snapshots at the end of the simulations showing (a) a fragmented and partially collapsed shell structure with $F^-_{NaCl(0.15M)}$ (b) the dispersed assembly with $H_2PO_4^-{}_{R2K}$.

Table S1. Overview of simulation conditions. "Counterions" are the ions required to neutralize the +1296 charges of the peptides. For systems with added salt, Na^+ and Cl^- ions were included in addition to the listed counterions, as indicated in the third column. Simulation lengths refer to the production runs following equilibration. Number of replicas denotes independent simulations under the same conditions.

System	Number of counterions (balancing peptide charges)	Added NaCl (concentration)	Simulation length (ns)	Number of replicas
$H_2PO_4^-$	1296	-	242/236	2
PO_4^{3-}	432	-	250/220	2
Cl^-	1296	-	238	1
F^-	1296	-	290	1
**Test on electrostatic screening**				
$F^-_{NaCl(1M)}$	1296	1 M	277	1
$F^-_{NaCl(0.15M)}$	1296	0.15 M	269	1
**Test on R2K mutation of CycVir**				
$H_2PO_4^-{}_{R2K}$	1296	-	250	1

Notes:

The Cl^-, F^-, and $H_2PO_4^-{}_{R2K}$ systems dispersed within 100 – 140 ns.

The $F^-_{NaCl(0.15M)}$ system formed a large hole on the shell within140 ns.

Table S2. Average number (rounded to the nearest integer) of hydrogen bonds between each counterion and peptides

Ion	Number of HBs
$H_2PO_4^-{}_{rep1}$	2 (81% to Arg)
$H_2PO_4^-{}_{rep2}$	2 (86% to Arg)
$PO_4^{3-}{}_{rep1}$	6 (100% to Arg)
$PO_4^{3-}{}_{rep2}$	6 (100% to Arg)

Table S3. Water coordination numbers of the first hydration shell to the anions in bulk solution and in the presence of peptides

Ion	Number of water oxygens with peptides	Distance of the 1st minimum from P/F/Cl
$H_2PO_4^-{}_{rep1}$	8	4.95 Å
$H_2PO_4^-{}_{rep2}$	8	4.95 Å
$PO_4^{3-}{}_{rep1}$	9	4.45 Å
$PO_4^{3-}{}_{rep2}$	9	4.45 Å

Table S4. Number of counterions condensed to the peptides, and number of counterions form HBs with side chains of Arg and Gln from two or more peptides.

Ion	Total	Ions condensed to the peptides	Ions bridging the peptides	Bridging $ratio_{total}$	Bridging $ratio_{condensed}$
$H_2PO_4^-{}_{rep1}$	1296	1165	372	29%	32%
$H_2PO_4^-{}_{rep2}$	1296	1170	366	28%	31%
$PO_4^{3-}{}_{rep1}$	432	432	406	94%	94%
$PO_4^{3-}{}_{rep2}$	432	431	410	95%	95%
